# An uncharacterized FMAG_01619 protein from *Fusobacterium mortiferum* ATCC 9817 demonstrates that some bacterial macrodomains can also act as poly-ADP-ribosylhydrolases

**DOI:** 10.1038/s41598-019-39691-4

**Published:** 2019-03-01

**Authors:** Antonio Ginés García-Saura, Rubén Zapata-Pérez, José Francisco Hidalgo, Juana Cabanes, Fernando Gil-Ortiz, Álvaro Sánchez-Ferrer

**Affiliations:** 1 Department of Biochemistry and Molecular Biology-A, Faculty of Biology, Regional Campus of International Excellence “Campus Mare Nostrum”, University of Murcia, Campus Espinardo, E-30100 Murcia, Spain; 2 Murcia Biomedical Research Institute (IMIB-Arrixaca), 30120 Murcia, Spain; 3 CELLS-ALBA Synchrotron Light Source, 08290 Barcelona, Spain

## Abstract

Macrodomains constitute a conserved fold widely distributed that is not only able to bind ADP-ribose in its free and protein-linked forms but also can catalyse the hydrolysis of the latter. They are involved in the regulation of important cellular processes, such as signalling, differentiation, proliferation and apoptosis, and in host-virus response, and for this, they are considered as promising therapeutic targets to slow tumour progression and viral pathogenesis. Although extensive work has been carried out with them, including their classification into six distinct phylogenetically clades, little is known on bacterial macrodomains, especially if these latter are able to remove poly(ADP-ribose) polymer (PAR) from PARylated proteins, activity that only has been confirmed in human TARG1 (C6orf130) protein. To extend this limited knowledge, we demonstrate, after a comprehensive bioinformatic and phylogenetic analysis, that *Fusobacterium mortiferum* ATCC 9817 TARG1 (FmTARG1) is the first bacterial macrodomain shown to have high catalytic efficiency towards O-acyl-ADP-ribose, even more than hTARG1, and towards mono- and poly(ADPribosyl)ated proteins. Surprisingly, FmTARG1 gene is also inserted into a unique operonic context, only shared by the distantly related *Fusobacterium perfoetens* ATCC 29250 macrodomain, which include an immunity protein 51 domain, typical of bacterial polymorphic toxin systems.

## Introduction

Macrodomains constitute a unique conserved 130–190 amino acids three-layered α/β/α sandwich fold widely distributed throughout the phylogenetic scale. They exert a regulatory influence on cellular signalling, transcription, DNA repair, genomic stability, telomere dynamics, necrosis and apoptosis, in addition to cell differentiation and proliferation^1^. They are also involved in different aspects of posttranslational protein modification of the ADP-ribosylation signalling pathway, including reading, erasing, and modulating, in combination with two other important NAD^+^-using protein families, poly(ADP-ribose) polymerases (PARPs) and sirtuins^2–9^. To carry out such a plethora of functions, macrodomains not only bind ADP-ribose (ADPr) in its free and protein-linked forms, but also related ligands, such as O-acyl-ADP-ribose (OAADPr), and even unconnected ones, such as poly(A) or G-quadruplex sequences^1^. In addition, they could also catalyse the hydrolysis of the 2′,1′′-O-glycosidic ribose-ribose bond in poly(ADP-ribose)-modified proteins (also called de-PARylation), the protein-mono-ADP-ribose (MAR) ester bond (de-MARylation), or the acyl-ADPr ester (deacylation)^10–14^.

Macrodomains have been phylogenetically subdivided into six distinct clades^1,14^. MacroD-type clade is the most studied and comprises several distinct proteins ranging from mono-(ADP-ribosyl) hydrolases and OAADPr deacetylases, such as human MacroD1 and MacroD2^15^, *Oceanobacillus iheyensis* MacroD (OiMacroD)^14^ and *E. coli* YmdB^16^, to poly(A) binding modules, such as GDAP2, a highly conserved protein found from plants to humans with a lipid-binding SEC14 domain at the C terminus^17^. Another class characterized by strong binding to ADP-ribosylated proteins without catalytic activity is the MacroH2A-type clade, which include the epigenetic tumour suppressor MacroH2A^18,19^, and the macrodomains from multidomain human PARP9, PARP14, and PARP15, which are involved in the regulation of cell migration associated with lymphoma (PARP9 and PARP14)^20^, in tumour suppression (PARP15)^21^ and in the host-virus response (PARP14 and PARP15)^22^. Macrodomain-containing proteins from C*oronaviridae*, *Togaviridae*, and *Hepeviridae* virus families have also been identified as a separate clade^23,24^, as a part of the multidomain nonstructural protein 3 (nsP3), which is involved in host protein recruitment and viral replication^25^. These macrodomains, also known as X domain or Mac1, are present in several (+)ssRNA viruses (e.g. hepatitis E virus, SARS coronavirus, human coronavirus HCoV 229E, Venezuelan equine encephalitis virus, and Chikungunya virus) and possess broad hydrolase activity towards mono-ADP-ribosylated proteins (PARP15, PARP14 and PARP10)^26^, but only weak activity towards PAR automodified PARP1^27^ and ADP-ribose-1′′-phosphate^28–30^. In addition, the nsP3 proteins of SARS coronavirus (SARS-CoV) and other β-coronaviruses (MERS-CoV, *BtVs-BetaCoV/SC2013*) contain a non-conserved region that follows the X domain (or Mac 1) with two additional macrodomains, Mac2 and Mac3, formerly known as SUD-N and SUD-M^25^, respectively. Mac2 and Mac3 do not bind or hydrolyse ADP-ribose, but do bind G quadruplexes^24,31^. However, while Mac2 has been seen to be dispensable for the SARS-CoV replication/transcription complex, Mac3 is essential for such processes, even though it is not conserved in all the coronaviruses^31^. Thus, viral macrodomains seem to be critical for viral pathogenesis and evasion of the host immune response, making them an important target for top-rated pathogens that are in need for vaccine development, such as Chikungunya virus^27^.

Poly(ADP-ribose) glycohydrolases (PARGs) form another diverged clade, which were recognized as macrodomains only after their seven-stranded mixed β-sheet sandwiched by a five α-helices structure with the PARG-specific GGGX_(6–8)_QEE catalytic motif within loop 1 was determined both in bacteria and human^32,33^. These enzymes release oligo- or poly(ADPr) fragments from PARylated proteins, being predominantly exoglycohydrolases with only minor contributions to debranching PAR polymers^34^. On the other hand, the Macro-2 type clade groups important and ubiquitous cellular processing macrodomain enzymes involved in the tRNA splicing pathway, which catalyses the conversion of ADP-ribose-1′′-phosphate (Appr-1′′-p) to ADP-ribose^35^, with no de-MARylating activity^36^. Their only studied representative is the hypothetical *Saccharomyces cerevisiae* ATCC 204508/S288c YMR087W protein (UniProt code YMX7_YEAST)^35^. By contrast, the YBR022W macrodomain protein of the same yeast strain (now named POA1) not only converts Appr-1′′-p to ADP-ribose, being responsible for ∼90% of Appr1p processing activity in yeast extracts, but also produces de-MARylation^37^. These differences in activity could be related to the fact that POA1 belongs to a different macrodomain clade, the ALC1 clade, thus named for the presence in it of the chromatin remodeler ALC1 (amplified in liver cancer 1) protein^38^, and whose macrodomain binds to sites of DNA damage by sensing PARP1-generated PAR^39^. Belonging to the same clade is the *Streptomyces coelicolor* SCO6735 macrodomain protein, which hydrolyses glutamate-linked protein mono-ADP-ribosylation, and whose depletion significantly increases the production of the antibiotic actinorhodin in *S. coelicolor*^40^. Interestingly, a unique class of macrodomains with DNA ADP-ribosyl glycohydrolase activity, such as *Mycobacterium tuberculosis* and *Thermus aquaticus* DarG proteins, could also be included in this clade. These last proteins reverse the thymidine base-specific ADP-ribosylation caused by the corresponding DarT toxins, a new class of DNA ADP-ribosyl transferases^41^. These DarG proteins are structurally most similar to metazoan TARG1 (terminal ADPr protein glycohydrolase1, also known as OARD1 or C6orf130) proteins, but with an active site mostly positively charged to facilitate the binding of their negatively charged ssDNA substrate^41^. Of note, full-length DarG seems to inhibit DarT through a protein-protein interaction, providing another layer of DarT regulation, in addition to its macrodomain hydrolytic activity^41^. This mechanism of DarT-DarG interaction has some parallelism with that recently described for the bifunctional immunity protein Tri1, an ADP-ribosyl hydrolase, which protects the essential bacterial tubulin-like protein FtsZ against the ADP-ribosylating toxin Tre1^42^.

Interestingly, the above-described human TARG1 (hTARG1) is the only known macrodomain able to hydrolyse OAADPr, MARylated proteins^10^, and the ribose-acceptor bond of PAR-modified PARP1, removing the entire PAR chain *en bloc*^12^. Its catalytic lysine residue forms a covalent intermediate with the ribose ring through an Amadori rearrangement mechanism, followed by aspartic acid-mediated hydrolysis^12^. Surprisingly, although this special class of macrodomain containing all three activities has been described in some bacteria as a possible alternative mechanism for reversing protein ADP-ribosylation^2^, they have not previously been characterized.

The present work presents a comprehensive bioinformatic and phylogenetic analysis of putative TARG1 proteins in bacterial organisms to better understand their distribution. We have focused our research on *Fusobacterium mortiferum* ATCC 9817 TARG1 because of its sequence identity with human TARG1, being the first protein ever characterised from this microorganism. Interestingly, we show that such an enzyme was the first bacterial macrodomain able to remove the PAR polymer, confirming this unique feature of TARG1-type macrodomains compared with MacroD-type macrodomains. In addition, the operonic structure in which FmTARG1 gene is inserted is also unique in nature and is only shared with the distantly related microorganism *F. perfoetens* ATCC 29250.

## Results

### TARG1-type proteins are scarce, representing just one percent of sequenced macrodomains

**Figure 1:**
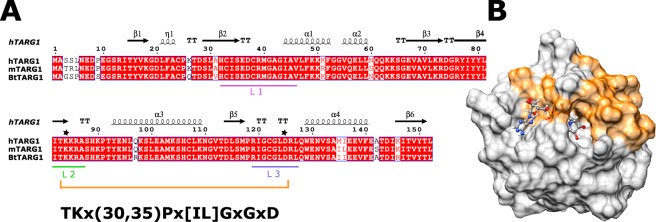
The TARG1-like pattern. (**A**) The structure-based sequence alignment of the eukaryotic revised TARG1-like macrodomain found in the UniProt database was carried out using ESPript^62^. The selected sequences were from human (hTARG1; Q9Y530), mouse (mTARG1; Q8R5F3) and bovine (BtTARG1; Q1LZ74). The catalytic residues K84 and D125 (hTARG1) are marked with black stars. The previously described ligand binding loops 1 to 3 are also underlined^12^. (**B**) The TARG1-like pattern described in this work is shown below the corresponding conserved region and coloured in orange on the hTARG1 surface structure (PDB code 4J5R).

**Figure 2:**
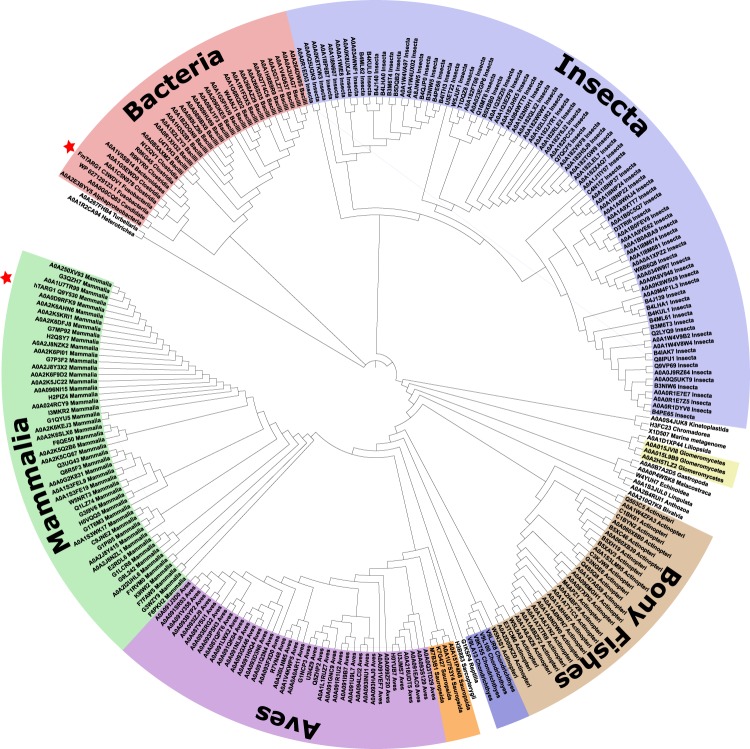
Phylogenetic analysis of TARG1-like proteins. The tree was constructed using the MAFFT neighbour-joining algorithm with 1000 replicates. Sequences are indicated by the UniProt code and the taxonomic Class level. Human and *Fusobacterium mortiferum* ATCC 9817 TARG1 representatives are marked with a red star. Eukaryotic sequences are summarized in Supplementary Table S1, whereas bacterial sequences are in Supplementary Table S2.

Human and reviewed mammalian TARG1 sequences were used to determine key sequence features to scan the UniProt database in an attempt to discover new canonical TARG1 members. These latter sequences are highly conserved, and their ADPr binding site can be structurally divided into three well defined regions (Fig. 1A). The first is where the adenosine moiety is bound by means of both the electrostatic interaction between the side chain carboxyl group of D20 (hTARG1 numbering) and the adenine primary amine, and the hydrophobic interactions with L21 and F22 on the β_1_-β_2_ loop, I44 and L47 on the β_2_-α_1_ loop (aka Loop1 or L1, Fig. 1A), and P118, Y150, and the C-terminal L152^10,12^. In the second region, the pyrophosphate moiety is embedded in the β_5_-α_4_ loop containing a GxG signature (L3, Fig. 1A), which is typical of phosphate-binding domains, and whose positive dipoles are oriented towards the phosphates to stabilize their negative charges^10,12^. Finally, the distal ribose region includes the vicinity of H32 and C33 main chain amides (L1, Fig. 1A), and the side chains of S35 (L1, Fig. 1A), T83 (L2, Fig. 1A), and D125 (L3, Fig. 1A), which possibly interact with its C2′′- and C3′′-hydroxyl groups^10,12^. Also in this distal ribose binding pocket is the catalytic residue, K84 (L2, Fig. 1A), which acts as a nucleophile to attack the C1″ ribose ring, forming a covalent lysyl-ADPr intermediate, which is decomposed via hydrolysis by D125^12^. Based on the relevance of Loop 2 and Loop 3 in the specific TARG1 catalytic mechanism, the Prosite-compatible pattern TKx(30,35)Px[IL]GxGxD was designed (Fig. 1A). This pattern covers the distance between T84 and D125, and basically represents the upper part of the binding site and the amino acids needed to obtain such a specific structure (Fig. 1B, tan surface). When this pattern was scanned against a macrodomain protein sequence database composed of 22435 sequences obtained using the Prosite pattern PS51154 (Domain Macro) in UniprotKB database (including Swiss-Prot and TrEMBL), only 326 sequences were found before curing them by length (130–230 residues), giving a total of 262 sequences. Among them, 87% were Eukaryotic (Fig. 2; Supplementary Table S1), and included the sequences of a spathe colourful flowering plant (*Anthurium amnicola* aka Tulip amnicola, Uniprot code A0A1D1XP44), a free-living nonparasitic kinetoplastid flagellated phagotrophic protozoan that feeds on bacteria (*Bodo saltans*, A0A0S4JUK8), a large trumpet ciliate protist (*Stentor coeruleus*, A0A1R2CA94), an arbuscular mycorrhizal fungus (*Rhizophagus irregularis*; A0A2H5TLZ2, A0A015JVI8, and A0A015L9B9) and 222 animals, among them, a cnidarian (*Stylophora pistillata*, A0A2B4RUI1). Surprisingly, insecta with 84 sequences is the most abundant group, and all of these sequences belong to the suborders Brachycera (58) and Climorpha (26), the latter suborder represented by a high number of mosquitos (24) that are vehicles of human diseases (Fig. 2). All chordata sequences belong to the Craniata subphylum, being almost uniformly distributed among Actinopteri (35, mainly bony fishes) and Aves (40) classes, but not in Reptilia, with only 5 sequences (Fig. 2). By contrast, the most abundant class is Mammalia with 50 sequences, of which, 27 are from Primates, including the one in human (Q9Y530) (Fig. 2).

**Figure 3:**
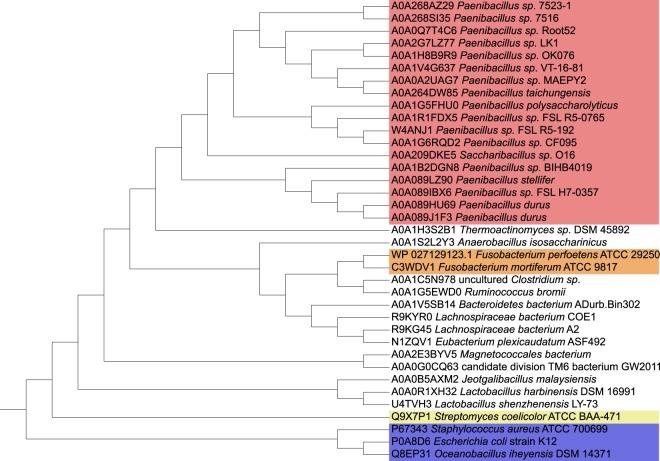
Phylogenetic analysis of bacterial TARG1-like macrodomains. The neighbour-joining tree was constructed using the 32 bacterial proteins that fulfil the TARG1 pattern, and another four bacterial macrodomains (one SCO6735-like and three MacroD-like) as outgroup. The tree was obtained after 1000 replicates.

Of note, TARG1 sequences were also found in Bacteria (33) (Fig. 2; Supplementary Table S2). Apart from one bacteroidete (A0A1V5SB14), an alphaproteobacteria (A0A2E3BYV5) and an unclassified metagenomic bacterium (A0A0G0CQ63), most of these sequences belong to Firmicutes (84.8%) and Fusobacteria (6.1%) phyla (Fig. 3). These sequences are mainly found in environmental or food samples, particularly those of *Paenibacillus* spp. In addition, one sequence was from rumen microbiome (A0A1G5EWD0), two from mouse ceca (N1ZQV1, R9KG45), two from faeces (A0A1C5N978, WP027129123.1) and one from a human infection (C3WDV1). The latter sequence, with a 61% sequence identity, belongs to *Fusobacterium mortiferum* ATCC 9817 (Supplementary Fig. S1), which was isolated from an abscess in the liver of a person who died at Johns Hopkins Hospital and was classified as *Bacillus mortiferus* in 1905^43^. *Fusobacterium mortiferum* has also been isolated from other human clinical specimens, such as colonic tissue from Crohn’s and Behcet’s disease patients^44^, maxillary abscesses (e.g. type strain ATCC 25557), and faeces (strains AM25-18LB, AM25-1 and OM06-15BH). Surprisingly, whereas several proteins from *F. mortiferum* ATCC 25557 have been studied^45–48^, mainly related to sugar metabolism, no proteins from *F. mortiferum* ATCC 9817 have ever been characterized.

**Figure 4:**
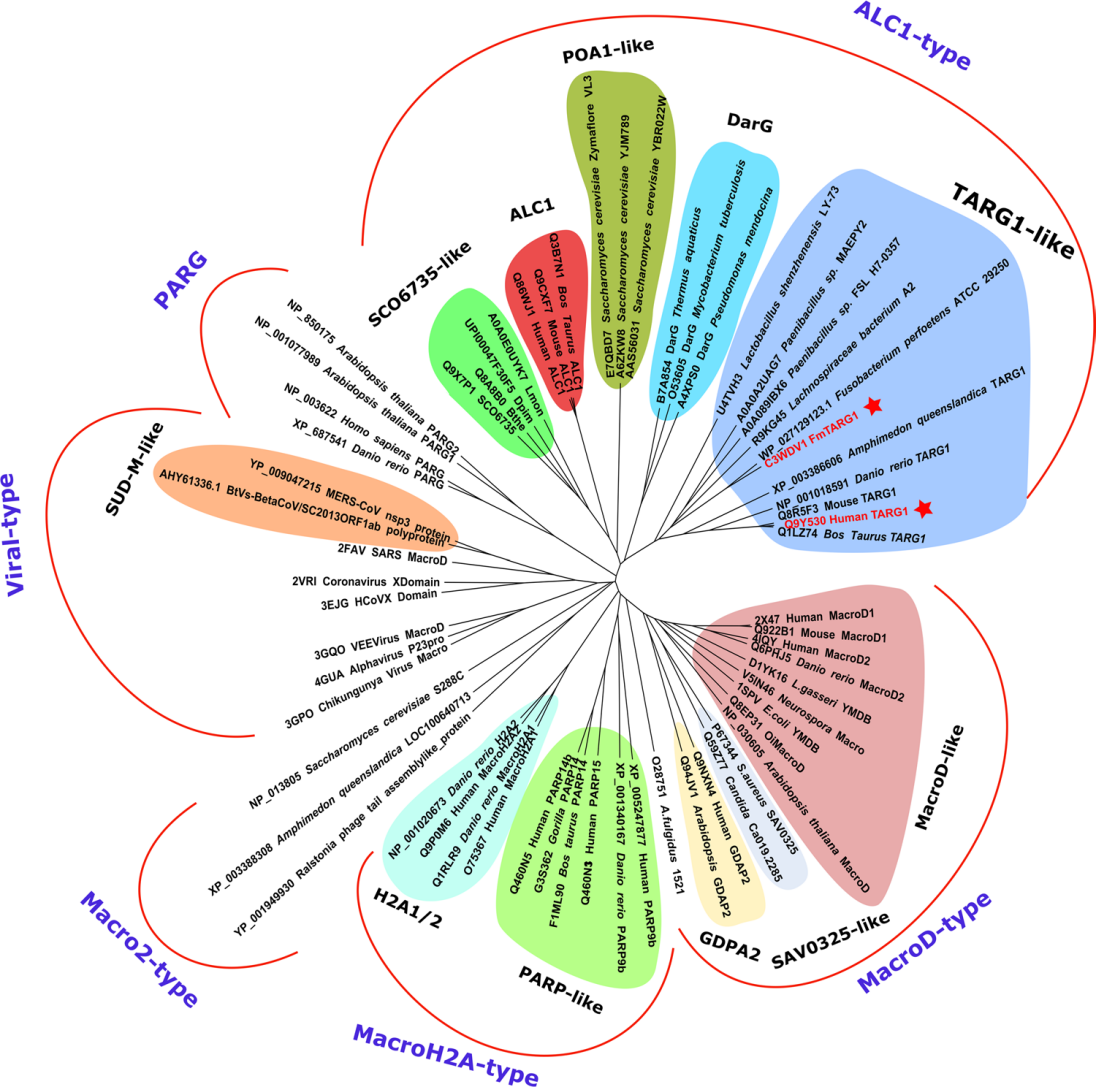
Macrodomain phylogenetic tree. The phylogram includes members of the six major macrodomain clades^1,14^. ALC1-type macrodomains could be divided in five subgroups, SCO6735-like, ALC1, POA1-like, DarG and TARG1-like. Human and *Fusobacterium mortiferum* ATCC 9817 TARG1 macrodomains are printed in red. The tree was obtained after 1000 replicates.

When representatives from the 33 bacterial TARG1 sequences were phylogenetically compared with other macrodomain sequences forming the six-clade tree (Fig. 4), a new and more accurate picture of the ALC1-type clade was observed than those previously described^1,14^. Thus, the ALC1-type clade is now divided into the five subclasses (Fig. 4), which include the SCO6735-like, the ALC1, the POA1-like, DarG and TARG1-like proteins. The first three subclasses appear to have a common phylogenetic origin, whereas the latter two form independent branches. Furthermore, in the case of the TARG1-like proteins there is a clear distinction between bacterial and eukaryotic sequences within the same clade. In addition, ALC1-type macrodomains appear to be widely distributed throughout the phylogenetic scale, except in the POA1-like subclass, which seems to be restricted to some *Saccharomyces cerevisiae* strains.

Apart from the ALC1-type clade, the updated sequences used in Fig. 4, also point to other subclasses in different clades. In the MacroD-type clade, four subclasses could be deciphered, starting from the most abundant, MacroD-like proteins, continuing with another consisting of operational macrodomains (MacroD and sirtuin-fused domains, SAV0325-like), another displaying poly(A) binding modules (GDPA2-like), and ending with the archaeal macrodomain of the Af1521 protein. MacroH2A-type is clearly divided into two branches, the H2A1/2 and the PARP-like, whereas Macro2-type and PARG are represented by uniform clades, with no clear subdivisions. However, viral macrodomains can be divided into two main subclasses, those belonging to *Togavirida*e and those to *Coraviridae*. This latter subclass also includes Mac3(SUD-M)-like macrodomains, which are able to bind G-quadruplex sequences^25,31^.

### *Fusobacterium* macrodomains are diverse, but TARG1-type genome context is unique

**Figure 5:**
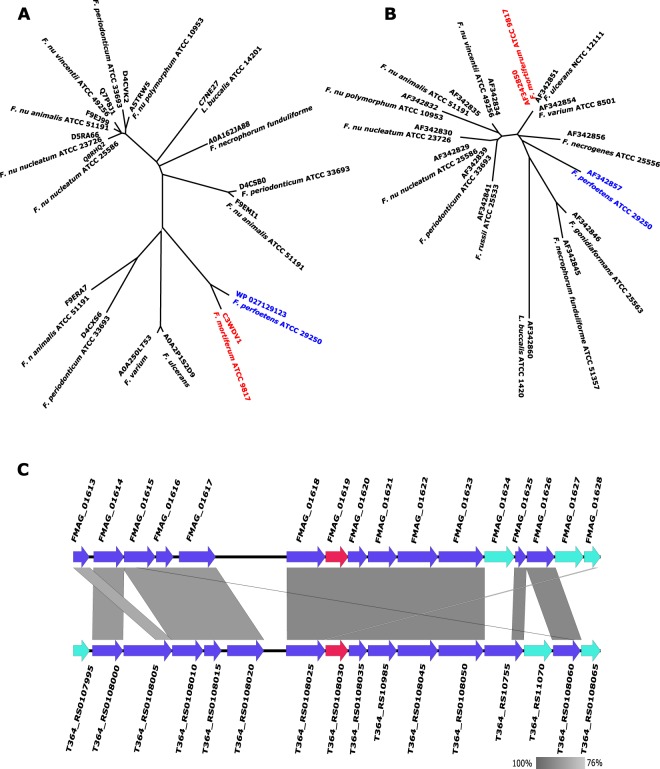
Distribution of *Fusobacterium* macrodomains. (**A**) Phylogenetic analysis of selected *Fusobacterium* macrodomains. *F. mortiferum* is highlighted in red and *F. perfoetens* in blue. (**B**) Phylogram showing the genetic relationships among fusobacterial species. Their short 16S–23S rDNA spacer region and that of *Leptotrichia buccalis* ATCC 14201 were used. Neihbour-joining trees were constructed using 1000 replicates. (**C**) Comparative genome map of *F. mortiferum* ATCC 9817 TARG1 gene (FMAG_01619) and the corresponding map in *F. perfoetens* ATCC 29250 (T364_RS0108030). Genes codifying the TARG1 proteins are marked in red. The figure was generated using EasyFig^61^ and shows the similarity between translated nucleotide sequences, as determined by the *tblastx* algorithm.

*F. mortiferum* ATCC 9817 TARG1 macrodomain (FmTARG1) is the only fusobacterial TARG1-type sequence found in UniProt. In order to explore similar sequences, we also scanned the NCBI database, finding only one additional protein (WP_027129123.1) in *F. perfoetens* ATCC 29250 (Fig. 5A; Supplementary Fig. S2), a strain that was isolated from piglet faeces^49^. These two sequences corresponded to only 2% of the macrodomains found in the genus (94). Most of them (84%) are MacroD-type macrodomains (e.g. F9EJ99 from *F. nucleatum subsp. animalis* ATCC 51191) (Fig. 5A; Supplementary Fig. S2) and include nine operonal macrodomains similar to SAV0325 protein (e.g. F9EMI1 from *F. nucleatum subsp. animalis* ATCC 51191) (Fig. 5A). In addition, there are also four sequences similar to SC06735 protein (e.g. A0A2P1S2D9 from *F. ulcerans*) (Fig. 5A; Supplementary Fig. S2) and nine similar to DarG macrodomains (e.g. F9ERA7 from *F. nucleatum subsp. animalis* ATCC 51191) (Fig. 5A; Supplementary Fig. S2). Of note is that some microorganisms have three subclasses of macrodomains (MacroD-like, SAV0325-like and DarG-like), including *F. nucleatum subsp. animalis* ATCC 51191 described above (Uniprot codes F9EJ99, F9EMI1 and F9ERA7) and *F. periodonticum* ATCC 33693 (UniProt codes D4CVK2, D4CSB0 and D4CXS6) (Fig. 5A; Supplementary Fig. S2).

This macrodomain distribution is clearly unrelated with *Fusobacterium* phylogeny (Fig. 5B), in light of the DNA sequences of their short 16S–23S rDNA spacer regions^50^, in which, an adaptive radiation into five clades has been found with an early point in its evolution from *Leptotrichia*^51^. *F. nucleatum* subspecies, along with *F. periodonticum* and *F. russii*, form clade 1, whereas *F. necrophorum* and *F. gonidiaformans* belong to clade 2. The third clade groups very closely related species from *F. mortiferum/varium/ulcerans* (93–98% similarity), which is also related with clade 4, which includes *F. necrogenes* (79–81%)^50^. Finally, *F. perfoetens*, known originally as *Coccobacillus perfoetens* for its distinct coccoid morphology, creates a quite separate clade from the other rod-shaped *Fusobacterium* species^51^.

Curiously, and although *F. mortiferum* ATCC 9817 and *F. perfoetens* ATCC 29250 belong to distantly related clades, their gene context analysis around TARG1 macrodomain genes (FMAG_01619 and T364_RS0108030, respectively) shows a similar operonic structure (Fig. 5C). In this structure, all the corresponding gene products are putative proteins with unknown functions (PFAM database DUFs numbers 1963, 2262, 4241, 4253, 4274, and 4299), except for the product of the contiguous gene FMAG_01620, in whose protein sequence an immunity protein 51 domain can be identified by PFAM. Proteins containing domains of this type are present in bacterial polymorphic toxin systems in the form of immediate gene neighbours of the toxin gene. These toxin operonic structures are present in some actinobacteria, bacteroidetes, β,γ-proteobacteria, cyanobacteria, spirochaetes, firmicutes and fusobacteria^52^. Importantly, this genome context is exclusive in nature to the two TARG1 macrodomains found in *Fusobacterium* spp., as the PATRIC database shows (https://www.patricbrc.org/). Such genome context singularity might be of biological relevance, although little is known about *F. mortiferum* biology. In fact, its strategy of invasion is different from that of other *Fusobacterium spp*., including ‘active’ and ‘passive’ invaders, since it lacks adhesion protein FadA and BMC (bacterial microcompartment) domains, and has a moderate number of MORN2 (membrane occupation and recognition nexus 2) domains^51^.

### FmTARG1 is the first bacterial macrodomain known to be capable of deacetylating, de-MARylating and de-PARylating ADPr-modified substrates

After sequence-based phylogenetic analysis, the *F. mortiferum* FMAG_01619 gene was cloned into the pSol-AFV vector and transformed into *E. cloni* 10G. After induction with L-rhamnose at 25 °C, the soluble recombinant protein was purified in three simple steps, as described in Materials and Methods. The purified monomeric enzyme (16820.32 Da) (Supplementary Fig. S3) showed deacetylase activity towards OAADPr and exhibited a saturation kinetics as its concentration increased up to 1 mM (Supplementary Fig. S4). The apparent K_M_ calculated at 25 °C for OAADPr was 145 ± 20 μM, with a *k*_cat_ of 0.64 ± 0.02 s^−1^ and a *k*_cat_/K_M_ of 4414 M^−1^ s^−1^ (Table 1). This latter catalytic efficiency is 1.4- to 2.4-fold higher than that described for other bacterial catalytic macrodomains (Table 1), such as EcYmdB (3042 M^−1^ s^−1^)^16^, OiMacroD (2412 M^−1^ s^−1^)^14^, and SaV0325 (1840 M^−1^ s^−1^)^15^, respectively. This difference in catalytic efficiency is much more pronounced compared with human macrodomains (Table 1), ranging from 2.6 to 8.3-fold in hTARG1 (1700 M^−1^ s^−1^)^10^, hMacroD2 (1100 M^−1^ s^−1^) and hMacroD1 (533 M^−1^ s^−1^)^15^, respectively. This variation was also maintained when hTARG1 cloned and purified as described in Materials and Methods was measured under the same experimental conditions used for FmTARG1 (1622 M^−1^ s^−1^) (Table 1).

**Table 1:** Comparison of kinetic parameters of FmTARG1 and described catalytic macrodomains towards OAADPr.

	*K*_M_ (µM)	*k*_cat_ (s^−1^)	*k*_cat_/*K*_M_ (M^−1^ s^−1^)
FmTARG1	145 ± 20	0.64 ± 0.02	4414
hTARG1^10^	182 ± 17	0.31 ± 0.03	1700
hTARG1	370 ± 25	0.60 ± 0.03	1622
OiMacro^14^	199 ± 23	0.48 ± 0.03	2412
EcYmdB^16^	430 ± 95	1.31 ± 0.12	3042
hMacroD1^15^	375 ± 55	0.20 ± 0.04	533
hMacroD2^15^	107 ± 38	0.12 ± 0.03	1100
SaV0325^15^	2000 ± 800	3.67 ± 1.22	1840

FmTARG1 also showed competitive inhibition by ADPr (Supplementary Fig. S4), as has been previously described for both hTARG1 and hMacroD1^10,15^. The inhibition constant (*K*_*i*_) obtained was 203 ± 10 μM, which is similar to that for hTARG1 and hMacroD1 (119 and 145 μM at 23 °C, respectively)^10,15^. These results indicate that FmTARG1 contains a specific binding site for ADPr, which is identical to the active site for OAADPr deacetylation. This could also be observed from the thermal shift assay carried out with both FmTARG1 and hTARG1 (Supplementary Fig. S5). ADPr and OAADPr protects FmTARG1 from thermal inactivation in a similar way, increasing its melting temperature (*T*_*m*_ = 52.5 ± 0.1 °C) by about 4.0 °C in a dose-dependent manner up to 1 mM (Supplementary Fig. S5A,B). This protection was higher, reaching up to 5.4 °C (Supplementary Fig. S5C,D) in hTARG1, whose melting temperature (*T*_*m*_ = 41.3 ± 0.2 °C) is about 10 °C lower than that of FmTARG1. In addition, ADPr analogues, such as AMP and ADP, also bind to both enzymes in a dose-dependent manner, but with lower affinities, since higher compound concentrations are needed (up to 50 mM) to observe similar thermal protection (Supplementary Fig. S5B,D). Collectively, these thermal shift experiments confirm that AMP is the smallest portion of the ADPr molecule that can bind to macrodomains not only in MacroD-type macrodomains^14^, but also in TARG1-type ones. In addition, it also reinforces the importance of the distal ribose in the binding of ADPr and OAADPr compared with ADP.

**Figure 6:**
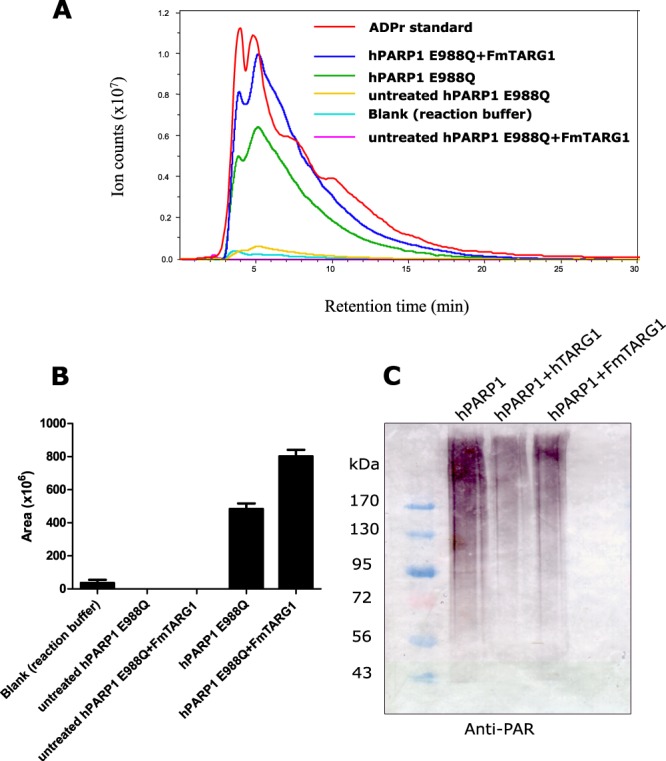
De-MARylation and de-PARylation activities of FmTARG1. (**A**) Ion chromatograms of the monoisotopic mass of the ADP-ribose [M + H]^+^ adduct (560 *m/z*) obtained after the mono-ADP-de-ribosylation reaction carried out by FmTARG1. (**B**) Area of mass 560.0 found in the different de-MARylation reactions. Error bars represent the standard deviation of three replicates. hPARP1 E988Q represents the mono-ADP-ribosylated protein, whereas untreated hPARP1 E988Q shows the unmodified protein. The blank is the PARP reaction buffer used to produce MARylated hPARP1 E988Q. (**C**) De-PARylation activities of FmTARG1 and hTARG1 carried out on auto-PARylated hPARP1 substrate. Numbers at the western blot margin indicate the molecular mass of the protein markers (kDa).

The ability of FmTARG1 to hydrolyse mono-ADP-ribosylated proteins *in vitro* was next tested using an automodified PARP1 E988Q mutant as a substrate and the reaction products were analysed by HPLC-ESI-MS/MS. Upon incubation of both proteins, a product that eluted at the same retention time as the ADP-ribose standard was detected (Fig. 6). This peak, and its corresponding area, was absent in the untreated PARP E988Q mutant (i.e. not automodified by the presence of NAD^+^ and activated DNA in the reaction medium), nor was it observed upon incubation of unmodified PARP with FmTARG1 (Fig. 6A,B). Thus, these results confirm that FmTARG1 removed ADPr from mono-ADP-ribosylated proteins, as previously described for other viral, ALC1-type, and MacroD-type macrodomains^12,14,24,31,37,40^.

Finally, the action of FmTARG1 towards poly(ADP-ribosyl)ated hPARP1 was further assayed in order to study its ability to remove PAR, as hTARG1 does^12,53^, and surprisingly, it was seen to be capable of hydrolysing the poly(ADP-ribose) polymer (Fig. 6C). However, its activity was lower than that observed with hTARG1 (Fig. 6C), which was also lower than that described for PARG^12^. In addition, when the reaction products of both enzymes (hTARG1 and FmTARG1) towards PARylated hPARP1were analysed by HPLC-ESI-MS/MS, the level of ADPr did not surpass that of PARylated hPARP1 in either case (Supplementary Fig. S6). These results reinforce the idea of FmTARG1 as a genuine bacterial hTARG1 analogue, sharing the ability to deacetylate, de-MARylate and de-PARylate ADPr-modified compounds (OAADPr) or proteins, making it the first bacterial macrodomain with this substrate profile to be described. In addition, it also confirms the existence of more macrodomains, other than hTARG1, able to carry out such plethora of activities.

## Discussion

Macrodomain proteins are involved in sensing, removing, and modulating reversible post-transcriptional modification reactions that involve NAD^+^ as substrate with the concomitant release of nicotinamide. These reactions are catalysed mainly by both a special class of glycosyltransferases, named ADP-ribosyltransferases (ARTs) that produce either the mono-ADP-ribosylation (MARylation) or the poly-ADP-ribosylation (PARylation) of their target proteins, and sirtuins, which generate protein deacetylation, rendering OAADPr. Although other enzymes, such as PARG and ADP-ribosylhydrolase 3 (ARH3), are also related with the PAR catabolism, among macrodomains, only TARG1-type macrodomains are capable of producing OAADPr deacetylation, de-MARylation and de-PARylation. Recently, hTARG1 has also been demonstrated to be able to reverse the ADP-ribosylation of DNA at both 5´P and 3´P ends, but its activity towards these DNA modified substrates was lower than that of ARH3, MacroD2, and PARG sequentially^54^. In addition, its role in the serine ADP-ribosylation posttranslational modification of proteins produced after DNA damage has been studied, showing that hTARG1 was not able to remove Ser-ADPr modification from a well-defined histone H3 peptide, whereas ARH3 was^55^.

These singular TARG1-type represented only 1% of total macrodomain sequences when protein databases were scanned using a profile based on the TARG1-type active centre (Fig. 1). These enzymes have mainly been found in Metazoa, with a few examples in Euglenozoa, Fungi, and Viridiplantae (Fig. 2). In addition, TARG1-like macrodomains were also detected in Bacteria (Fig. 3), where they represented only 13% of the 262 TARG1-like sequences selected (Fig. 2), forming a defined branch in the six-clade macrodomain phylogenetic tree (Fig. 4). Among these sequences, only one belongs to a microorganism (*F. mortiferum* ATCC 9817) that infects human, whereas the rest of the sequences come from microorganisms retrieved from environmental samples or animal faeces. Curiously, the above microorganism has a very limited PAR metabolism, comprising just one TARG1-like macrodomain (C3WDV1) and one Sir2(CobB) protein (C3WDZ8), without any PARP, PARG or ARH3-like enzymes. In addition, its corresponding macrodomain gene (*FMAG_01619*) is inserted in a unique operonic structure (Fig. 5), which is only shared with the *F. perfoetens* macrodomain gene (*T364_RS0108030*), and formed basically of genes whose protein products are unknown, except for one with an immunity 51 domain. This last domain is related with bacterial polymorphic toxins (Fig. 5C). Interestingly, this scheme (only one macrodomain) is not generally applicable to other *Fusobacterium* spp., since, and unexpectedly, some of them (e.g. *F. nucleatum subsp. animalis* ATCC 51191 and *F. periodonticum* ATCC 33693) display several macrodomains, each of them from a different clade (MacroD-, SAV0325- and DarG-like, respectively) (Fig. 5A). Kinetically, *F. mortiferum* ATCC 9817 TARG1 macrodomain (FmTARG1) is a remarkable catalyst, since it showed the highest catalytic efficiency described for a macrodomain, 2.6-fold higher than its homologous human enzyme (hTARG1) and 8.3-fold higher than hMacroD1 (Table 1; Supplementary Fig. S4)^10,15^. Of note too is the fact that FmTARG1 was not only capable of removing terminal ADPr from MARylated proteins (Fig. 6A,B), but also PAR from PARylated hPARP1 (Fig. 6C), confirming that is a canonical TARG1-type macrodomain and that hTARG1 is not the only enzyme with these three activities. This, together with its high thermal stability compared with hTARG1 (Supplementary Fig. S4) and some MacroD proteins (e.g. OiMacroD), makes FmTARG1 an ideal biocatalyst for obtaining PAR polymer from PARylated proteins in order to study relevant molecular processes in which PAR is involved, such as Parthanatos^56^. Finally, in *F. mortiferum* ATCC 9817 TARG1-type macrodomain could be associated with the forefront ADP-ribosylation interaction between the bacterium and the host, mediated by PARP enzymes. In addition, it is tempting to speculate that some of the products of its operonic structure might also be related with this host-pathogen response. Finally, the above-described results will provide the foundation for a search for new future bacterial macrodomains from the vast number of sequences described in the databases, which will expand our knowledge of the PAR metabolism.

## Materials and Methods

### Protein expression and purification

*Fusobacterium mortiferum* ATCC 9817 TARG1 macrodomain (Uniprot code, C3WDV1) was cloned into the pSol-AFV vector (Lucigen, USA), using the synthetic *FMAG_01619* gene obtained from Genscript (USA), and transformed into *Escherichia coli* 10G^57^. The clone containing the FmTARG1 was grown in 1 L of Terrific Broth (TB) medium supplemented with kanamycin (50 µg mL^−1^). When the culture reached an optical density of 4.5 at 600 nm, it was induced with 0.2% L-rhamnose for 16 h at 25 °C. The culture was centrifuged, and the pellet resuspended in lysis buffer (20 mM Bis-Tris pH 7.5, 300 mM NaCl). Cells were disrupted using a Bead Beater homogenizer (Biospec). After ultracentrifugation (40000 *g*, 40 min), the supernatant was loaded at 4 °C onto a HiPrep IMAC 16/10 FF column (GE Lifesciences, Spain) coupled to a FPLC chromatography system (ÄKTA Prime Plus, GE Lifesciences). The protein containing FmTARG1 fractions were pooled and desalted, before removing AFV-tag with a 6His-tag TEV protease. After a new chromatographic step onto the HisTrap FF column, the pure protein was loaded into a Superdex 200 HiLoad 16/600 column (GE Lifesciences), obtaining an electrophoretically pure enzyme. In addition, the molecular mass of highly pure FmTARG1 was determined using an Agilent 1290 Infinity II Series HPLC coupled to an Agilent 6550 Q-TOF Mass Spectrometer^58^. FmTARG1was stored at −20 °C with 10% glycerol.

Human TARG1 (Uniprot code Q9Y530) was also cloned using its corresponding synthetic gene, but inserted into pET28a vector (Merck, Spain), transformed into *E. coli* Rosetta2(DE3) and induced using 0.2 mM isopropyl-β-D-thiogalactoside (IPTG) for 16 h at 20 °C with constant shaking. In this case, the purification protocol was the same as above except for using a single Ni^2+^-chelating affinity chromatographic step. The protein concentration was determined using Bradford reagent (Bio-Rad, Spain) and BSA as standard.

### Deacetylation assay

Macrodomain deacetylating activity was followed by HPLC using OAADPr as substrate at 25 °C. The reaction medium (250 µL) contained 20 mM Bis-Tris buffer pH 7.3 with 300 mM NaCl, different concentrations of OAADPr (25–1000 µM) and 0.2 µM FmTARG1 or hTARG1. Reactions were stopped with 1% TFA (v/v) and then injected into on HPLC apparatus (Agilent 1100 series) with a C18 column (Gemini C18, 4.6 × 250 mm, Phenomenex) and mobile phase running at 1 mL min^−1^, following a linear gradient for 12 min from 0% MilliQ water with 0.015% TFA (solvent A) to 8% acetonitrile with 0.02% TFA (solvent B)^14^. The reactions were carried out in triplicate and their corresponding SD values were calculated. Commercial OAADPr (TRC, Canada) was used as a standard.

### De-MARylation assay

Human PARP1 E988Q mutant, an accepted model of mono(ADP-ribosyl)ated PARP1 substrate^32^, was prepared by mutating the hPARP1vector (kindly provided by J. M. Pascal, Université de Montréal, Montréal, QC), as previously described^14^. NAD^+^-dependant automodification of hPARP1 E988Q mutant (2.1 μM) was carried out using PARP reaction buffer (100 mM Tris-HCl pH 8.0, 10 mM MgCl_2_ and 1 mM DTT), 200 μM β-NAD^+^ (Trevigen) and 17 μg activated DNA (Trevigen, UK) for 1 hour at 30 °C. The reaction was finished by adding Rupacarib (5 μM), a well-known PARP1 inhibitor. To the latter solution, FmTARG1 (8 μM) was added and incubated for 30 minutes at 25 °C, before placing it on ice. The released ADP-ribose was purified by ultrafiltration over Amicon Ultracel-3 column^59^, before analysis on an HPLC-MS equipment consisting of an Agilent 1100 Series HPLC coupled to an Agilent Ion Trap XCT Plus Mass Spectrometer using an electrospray (ESI) interface. Each sample (40 μL) was subjected to reversed-phase liquid chromatography on a C18 column (Discovery, 100 × 2.1 mm, Supelco) and eluted at 0.2 mL min^−1^ with a mobile phase A (0.05% TFA in MilliQ^®^ water) and B (0.05% TFA in acetonitrile) 90:10 (v/v) for 10 minutes followed by a linear gradient from 10 to 100% solvent B in 20 minutes. The column was re-equilibrated with mobile phase A for 10 minutes before a new run. The mass spectrometer was operated in the positive mode with a capillary spray voltage of 3.5 kV. Data were obtained in the MS and MS/MS mode using MRM and processed using the DataAnalysis program for LC/MSD Trap Version 3.2 provided by the manufacturer. ADPr was detected as the [M + H]^+^ ion at 560 *m/z* and confirmed with the transition 560 > 348 *m/z*. All the experiments were carried out in triplicate.

### De-PARylation assay

TARG1-type macrodomain PAR hydrolysis was determined by western blot^33^. The substrate used was automodified hPARP obtained as described above using NAD^+^ and activated DNA as substrates and Rucaparib to stop the reaction. Then, this PARylated substrate was incubated for 1 hour at 30 °C in the presence of FmTARG1 (20 μM) or hTARG1 (10 μM), before running the samples on 7–10% SDS-PAGE gels and transfering to a nitrocellulose membrane. The presence of PAR was detected by coupling with rabbit polyclonal anti-PAR antibodies (1:1000, Trevigen) and goat-anti-rabbit-HRP conjugated secondary antibody (Bio-Rad), and finally revealed with Opti-4CN substrate (Bio-Rad) for 5 minutes. Analysis of the reaction products of hTARG1 and FmTARG1 on poly (ADP-ribosyl)ated PARP1 was carried out by mass spectrometry as described above for the de-MARylation assay.

### Thermal stability assay

Protein melting curves of FmTARG1 and hTARG1 were obtained in 7500 RT-PCR equipment (Applied Biosystems) using the 10X fluorescent dye SYPRO Orange (Molecular Probes), as previous described^60^. Both enzymes (10 µg) were preincubated in triplicate in the presence of 100 mM phosphate buffer pH 7.0 and titrated with four different ligands (AMP, ADP, ADPr or OAADPr). The samples were slowly heated from 20 to 80 °C and the fluorescence was recorded every 1 °C at 530 nm.

### Bioinformatic analysis

Protein sequences were obtained from NCBI non-redundant (NR) and UniProt databases. Incomplete sequences and duplicates were removed, rendering the sequences described in Supplementary Tables S1 and S2. Neighbour-Joining (NJ) trees with 1000 replicates were constructed using the MAFFT server (https://mafft.cbrc.jp/alignment/server/) and visualized with iTOL (https://itol.embl.de/). Genome alignments were performed using EasyFig^61^.

## Supplementary information

Supplementary info
