# Supplementary material for: An uncharacterized FMAG_01619 protein from Fusobacterium mortiferum ATCC 9817 demonstrates that some bacterial macrodomains can also act as poly-ADP-ribosylhydrolases
Source: Sci Rep. 2019 Mar 1;9:3230. doi: 10.1038/s41598-019-39691-4 (PMC6397177; doi:10.1038/s41598-019-39691-4)
Supplement: Supplementary file 1 — Supplementary info [file 41598_2019_39691_MOESM1_ESM.pdf]

Supplementary Information

An uncharacterized FMAG_01619 protein from *Fusobacterium mortiferum* ATCC 9817 demonstrates that some bacterial macrodomains can also act as poly-ADP-ribosylhydrolases

Authors:

Antonio Ginés García-Saura[1,2], Rubén Zapata-Pérez[1,2], José Francisco-Hidalgo[1], Juana Cabanes[1,2], Fernando Gil-Ortiz[3] , & Álvaro Sánchez-Ferrer[1,2,*]

Affiliations:

[1]Department of Biochemistry and Molecular Biology-A, Faculty of Biology, Regional Campus of International Excellence "Campus Mare Nostrum", University of Murcia, Campus Espinardo, E-30100 Murcia, Spain.

[2]Murcia Biomedical Research Institute (IMIB-Arrixaca), 30120 Murcia, Spain.

[3]CELLS-ALBA Synchrotron Light Source, 08290 Barcelona, Spain.

*Corresponding author A.S-F.; alvaro@um.es.

UniProt code	Superkingdom	Class	UniProt code	Superkingdom	Class	UniProt code	Superkingdom	Class
A0A015JVI8	Eukaryota	Glomeromycetes	A0A182XAQ7	Eukaryota	Insecta	B4KUL0	Eukaryota	Insecta
A0A015L9B9	Eukaryota	Glomeromycetes	A0A182Y6J2	Eukaryota	Insecta	B4KUL1	Eukaryota	Insecta
A0A023EGP6	Eukaryota	Insecta	A0A1A7Y1C5	Eukaryota	Actinopteri	B4LHA0	Eukaryota	Insecta
A0A024RCY9	Eukaryota	Mammalia	A0A1A7ZTR8	Eukaryota	Actinopteri	B4LHA1	Eukaryota	Insecta
A0A034W9I7	Eukaryota	Insecta	A0A1A8BZF2	Eukaryota	Actinopteri	B4ML61	Eukaryota	Insecta
A0A034WNF1	Eukaryota	Insecta	A0A1A8H487	Eukaryota	Actinopteri	B4ML62	Eukaryota	Insecta
A0A060X8B0	Eukaryota	Actinopteri	A0A1A8J6Z8	Eukaryota	Actinopteri	B4PE65	Eukaryota	Insecta
A0A060XB39	Eukaryota	Actinopteri	A0A1A8JYN2	Eukaryota	Actinopteri	B4PE66	Eukaryota	Insecta
A0A084WTT0	Eukaryota	Insecta	A0A1A8NDW0	Eukaryota	Actinopteri	B4QJP0	Eukaryota	Insecta
A0A087QPT6	Eukaryota	Aves	A0A1A8NPN7	Eukaryota	Actinopteri	B5DQB2	Eukaryota	Insecta
A0A087V5U1	Eukaryota	Aves	A0A1A9VE62	Eukaryota	Insecta	B5XAV3	Eukaryota	Actinopteri
A0A087XPI2	Eukaryota	Actinopteri	A0A1A9WHJ4	Eukaryota	Insecta	B5XC46	Eukaryota	Actinopteri
A0A091EAC0	Eukaryota	Aves	A0A1A9XTT7	Eukaryota	Insecta	B5XH19	Eukaryota	Actinopteri
A0A091GNC9	Eukaryota	Aves	A0A1B0ABA9	Eukaryota	Insecta	C1BK61	Eukaryota	Actinopteri
A0A091H0Q4	Eukaryota	Aves	A0A1B0C5Q7	Eukaryota	Insecta	C1BYN2	Eukaryota	Actinopteri
A0A091I9B7	Eukaryota	Aves	A0A1B0FEV8	Eukaryota	Insecta	C3KJW8	Eukaryota	Actinopteri
A0A091J3D0	Eukaryota	Aves	A0A1D1XP44	Eukaryota	Liliopsida	C4N157	Eukaryota	Insecta
A0A091LWX2	Eukaryota	Aves	A0A1I8M674	Eukaryota	Insecta	C9JNE2	Eukaryota	Mammalia
A0A091NLI8	Eukaryota	Aves	A0A1I8M681	Eukaryota	Insecta	D3TRI6	Eukaryota	Insecta
A0A091Q3N4	Eukaryota	Aves	A0A1I8N607	Eukaryota	Insecta	E2RDL6	Eukaryota	Mammalia
A0A091QK04	Eukaryota	Aves	A0A1I8NP22	Eukaryota	Insecta	E3TCM6	Eukaryota	Actinopteri
A0A091R1U2	Eukaryota	Aves	A0A1I8NP24	Eukaryota	Insecta	F1RVM0	Eukaryota	Mammalia
A0A091SR03	Eukaryota	Aves	A0A1I8NP37	Eukaryota	Insecta	F6PKH2	Eukaryota	Mammalia
A0A091U9L7	Eukaryota	Aves	A0A1I8P6B7	Eukaryota	Insecta	F6QE50	Eukaryota	Mammalia
A0A091V358	Eukaryota	Aves	A0A1J1IY07	Eukaryota	Insecta	F7FAW5	Eukaryota	Mammalia
A0A091VEF7	Eukaryota	Aves	A0A1L1RUZ7	Eukaryota	Aves	G1KSP4	Eukaryota	*Reptilia*
A0A093BYP2	Eukaryota	Aves	A0A1Q3EZF9	Eukaryota	Insecta	G1LCR6	Eukaryota	Mammalia
A0A093D3N6	Eukaryota	Aves	A0A1Q3EZI5	Eukaryota	Insecta	G1NCP3	Eukaryota	Aves
A0A093ERG2	Eukaryota	Aves	A0A1R2CA94	Eukaryota	Heterotrichea	G1PI05	Eukaryota	Mammalia
A0A093FXD0	Eukaryota	Aves	A0A1S3FE19	Eukaryota	Mammalia	G1QYU5	Eukaryota	Mammalia
A0A093GX46	Eukaryota	Aves	A0A1S3FEL9	Eukaryota	Mammalia	G1T6M3	Eukaryota	Mammalia
A0A093HAJ0	Eukaryota	Aves	A0A1S3JUL0	Eukaryota	Lingulata	G3I9V6	Eukaryota	Mammalia
A0A093INU1	Eukaryota	Aves	A0A1S3LM95	Eukaryota	Actinopteri	G3NGE6	Eukaryota	Actinopteri
A0A093IZJ9	Eukaryota	Aves	A0A1S3WK17	Eukaryota	Mammalia	G3QZH7	Eukaryota	Mammalia
A0A093P5H3	Eukaryota	Aves	A0A1U7S3Y4	Eukaryota	*Sauropsida*	G3WZY9	Eukaryota	Mammalia
A0A093S129	Eukaryota	Aves	A0A1U7TR99	Eukaryota	Mammalia	G7MP92	Eukaryota	Mammalia
A0A094LCI2	Eukaryota	Aves	A0A1V4KWP5	Eukaryota	Aves	G7P3F2	Eukaryota	Mammalia
A0A096MGH1	Eukaryota	Actinopteri	A0A1W4UX02	Eukaryota	Insecta	G9L242	Eukaryota	Mammalia
A0A096NI15	Eukaryota	Mammalia	A0A1W4V8W4	Eukaryota	Insecta	H0VQQ5	Eukaryota	Mammalia
A0A099ZF20	Eukaryota	Aves	A0A1W4V9B2	Eukaryota	Insecta	H0YUB7	Eukaryota	Aves
A0A0A0AR11	Eukaryota	Aves	A0A1W4VA97	Eukaryota	Insecta	H2PIZ4	Eukaryota	Mammalia

A0A0A1WIE8	Eukaryota	Insecta	A0A1W4ZFA3	Eukaryota	Actinopteri	H2QSY7	Eukaryota	Mammalia
A0A0A1XPZ2	Eukaryota	Insecta	A0A210Q7K8	Eukaryota	Bivalvia	H3B883	Eukaryota	*Sarcopterygii*
A0A0B7A2D5	Eukaryota	Gastropoda	A0A218UDT5	Eukaryota	Aves	H3FC23	Eukaryota	Chromadorea
A0A0D9RFK9	Eukaryota	Mammalia	A0A250XV93	Eukaryota	Mammalia	I3MKR2	Eukaryota	Mammalia
A0A0G2K831	Eukaryota	Mammalia	A0A267FHB4	Eukaryota	*Turbellaria*	K7G427	Eukaryota	*Sauropsida*
A0A0J9RZ64	Eukaryota	Insecta	A0A2B4RUI1	Eukaryota	Anthozoa	K9IWI2	Eukaryota	Mammalia
A0A0K8TKW3	Eukaryota	Insecta	A0A2D0SKZ0	Eukaryota	Actinopteri	M4APZ1	Eukaryota	Actinopteri
A0A0K8UXJ4	Eukaryota	Insecta	A0A2H5TLZ2	Eukaryota	Glomeromycetes	M7B3B1	Eukaryota	*Sauropsida*
A0A0K8V948	Eukaryota	Insecta	A0A2I0LNM5	Eukaryota	Aves	Q16MT6	Eukaryota	Insecta
A0A0K8W5U9	Eukaryota	Insecta	A0A2I2UHL6	Eukaryota	Mammalia	Q1LZ74	Eukaryota	Mammalia
A0A0M4F1L3	Eukaryota	Insecta	A0A2I4BF28	Eukaryota	Actinopteri	Q2LYQ9	Eukaryota	Insecta
A0A0P4WSK8	Eukaryota	Malacostraca	A0A2J8NZK2	Eukaryota	Mammalia	Q3UG43	Eukaryota	Mammalia
A0A0Q3TD29	Eukaryota	Aves	A0A2J8NZL1	Eukaryota	Mammalia	Q4S5Q8	Eukaryota	Actinopteri
A0A0Q5UGK9	Eukaryota	Insecta	A0A2J8Y3X2	Eukaryota	Mammalia	Q503C5	Eukaryota	Actinopteri
A0A0Q5UKT9	Eukaryota	Insecta	A0A2J8Y415	Eukaryota	Mammalia	Q5ZHP2	Eukaryota	Aves
A0A0R1DYV8	Eukaryota	Insecta	A0A2K5CG67	Eukaryota	Mammalia	Q7QCF5	Eukaryota	Insecta
A0A0R1E7E7	Eukaryota	Insecta	A0A2K5JC22	Eukaryota	Mammalia	Q8IPU1	Eukaryota	Insecta
A0A0R1E7Z5	Eukaryota	Insecta	A0A2K5KRI1	Eukaryota	Mammalia	Q8R5F3	Eukaryota	Mammalia
A0A0R1EDI3	Eukaryota	Insecta	A0A2K5Q2B6	Eukaryota	Mammalia	Q9VP69	Eukaryota	Insecta
A0A0S4JUK8	Eukaryota	*Kinetoplastida*	A0A2K6AHN6	Eukaryota	Mammalia	Q9Y530	Eukaryota	Mammalia
A0A147A659	Eukaryota	Actinopteri	A0A2K6DFJ8	Eukaryota	Mammalia	R7VN48	Eukaryota	Aves
A0A151P4N8	Eukaryota	*Sauropsida*	A0A2K6F9D2	Eukaryota	Mammalia	T1DQZ8	Eukaryota	Insecta
A0A182FT06	Eukaryota	Insecta	A0A2K6KEJ3	Eukaryota	Mammalia	U3I4Z9	Eukaryota	Aves
A0A182HSJ9	Eukaryota	Insecta	A0A2K6PI01	Eukaryota	Mammalia	U3JMS7	Eukaryota	Aves
A0A182JHK5	Eukaryota	Insecta	A0A2K6SLX6	Eukaryota	Mammalia	U5ET22	Eukaryota	Insecta
A0A182JYR1	Eukaryota	Insecta	A8JNW5	Eukaryota	Insecta	V9KT55	Eukaryota	Chondrichthyes
A0A182LEL7	Eukaryota	Insecta	B0XBB0	Eukaryota	Insecta	V9L100	Eukaryota	Chondrichthyes
A0A182LUR2	Eukaryota	Insecta	B3M6T3	Eukaryota	Insecta	V9LA13	Eukaryota	Chondrichthyes
A0A182NRH1	Eukaryota	Insecta	B3M6T4	Eukaryota	Insecta	V9LJR8	Eukaryota	Chondrichthyes
A0A182PCC8	Eukaryota	Insecta	B3NIW5	Eukaryota	Insecta	W4YUH7	Eukaryota	Echinoidea
A0A182QLX2	Eukaryota	Insecta	B3NIW6	Eukaryota	Insecta	W5JUF1	Eukaryota	Insecta
A0A182RLE2	Eukaryota	Insecta	B4IAK7	Eukaryota	Insecta	W5L9A9	Eukaryota	Actinopteri
A0A182TGW9	Eukaryota	Insecta	B4ITH3	Eukaryota	Insecta	W5N3S1	Eukaryota	Actinopteri
A0A182VKF8	Eukaryota	Insecta	B4J139	Eukaryota	Insecta	W5NRT3	Eukaryota	Mammalia
A0A182W0W2	Eukaryota	Insecta	B4J140	Eukaryota	Insecta	W8B6Q8	Eukaryota	Insecta

Supplementary Table S1. Eukaryotic TARG1-like sequences found in NCBI non-redundant (NR) and UniProt databases. These sequences were used in the phylogenetic analysis of Fig. 2. Sequences in italics represent the taxonomic level of Order.

UniProt code	Superkingdom	Class	species
A0A089HU69	Bacteria	Bacilli	*Paenibacillus durus*
A0A089IBX6	Bacteria	Bacilli	*Paenibacillus sp.* FSL H7-0357
A0A089J1F3	Bacteria	Bacilli	*Paenibacillus durus*
A0A089LZ90	Bacteria	Bacilli	*Paenibacillus stellifer*
A0A0A2UAG7	Bacteria	Bacilli	*Paenibacillus sp.* MAEPY2
A0A0B5AXM2	Bacteria	Bacilli	*Jeotgalibacillus malaysiensis*
A0A0G0CQ63	Bacteria	Candidatus	*candidate division* TM6 bacterium GW2011_GWF2_33_332
A0A0Q7T4C6	Bacteria	Bacilli	*Paenibacillus sp.* Root52
A0A0R1XH32	Bacteria	Bacilli	*Lactobacillus harbinensis* DSM 16991
A0A1B2DGN8	Bacteria	Bacilli	*Paenibacillus sp.* BIHB4019
A0A1C5N978	Bacteria	Clostridia	*uncultured Clostridium sp.*
A0A1G5EWD0	Bacteria	Clostridia	*Ruminococcus bromii*
A0A1G5FHU0	Bacteria	Bacilli	*Paenibacillus polysaccharolyticus*
A0A1G6RQD2	Bacteria	Bacilli	*Paenibacillus sp.* CF095
A0A1H3S2B1	Bacteria	Bacilli	*Thermoactinomyces sp.* DSM 45892
A0A1H8B9R9	Bacteria	Bacilli	*Paenibacillus sp.* OK076
A0A1R1FDX5	Bacteria	Bacilli	*Paenibacillus sp.* FSL R5-0765
A0A1S2L2Y3	Bacteria	Bacilli	*Anaerobacillus sp.* NB2006
A0A1V4G637	Bacteria	Bacilli	*Paenibacillus sp.* VT-16-81
A0A1V5SB14	Bacteria	Bacteroidetes	*Bacteroidetes bacterium* ADurb.Bin302
A0A209DKE5	Bacteria	Bacilli	*Saccharibacillus sp.* O16
A0A264DW85	Bacteria	Bacilli	*Paenibacillus taichungensis*
A0A268AZ29	Bacteria	Bacilli	*Paenibacillus sp.* 7523-1
A0A268SI35	Bacteria	Bacilli	*Paenibacillus sp.* 7516
A0A2E3BYV5	Bacteria	Alphaproteobacteria	*Magnetococcales bacterium*
A0A2G7LZ77	Bacteria	Bacilli	*Paenibacillus sp.* LK1
C3WDV1	Bacteria	Fusobacteriia	*Fusobacterium mortiferum* ATCC 9817
N1ZQV1	Bacteria	Clostridia	*Eubacterium plexicaudatum* ASF492
R9KG45	Bacteria	Clostridia	*Lachnospiraceae bacterium* A2
R9KYR0	Bacteria	Clostridia	*Lachnospiraceae bacterium* COE1
U4TVH3	Bacteria	Bacilli	*Lactobacillus shenzhenensis* LY-73
W4ANJ1	Bacteria	Bacilli	*Paenibacillus sp.* FSL R5-192
WP_027129123	Bacteria	Fusobacteriia	*Fusobacterium perfoetens*

Supplementary Table S2. Bacterial TARG1-like sequences found in NCBI non-redundant (NR) and UniProt databases. These sequences were used in the phylogenetic analysis of Fig. 3. Sequences underlined represent not fully classified bacteria.

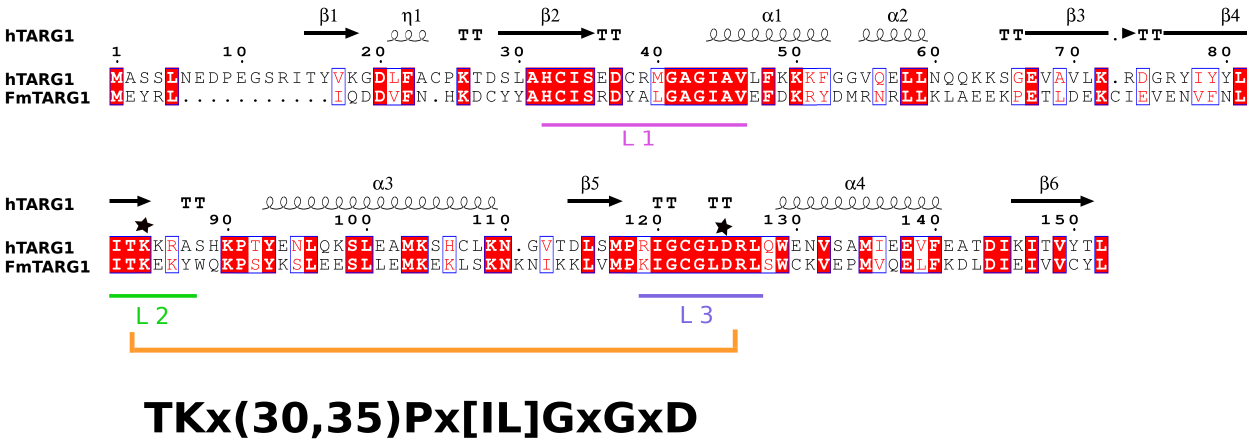

Supplementary Figure S1. The structure-based sequence alignment of FmTARG1 and hTARG1 sequences. The symbols and legends are the same as in Fig. 1.

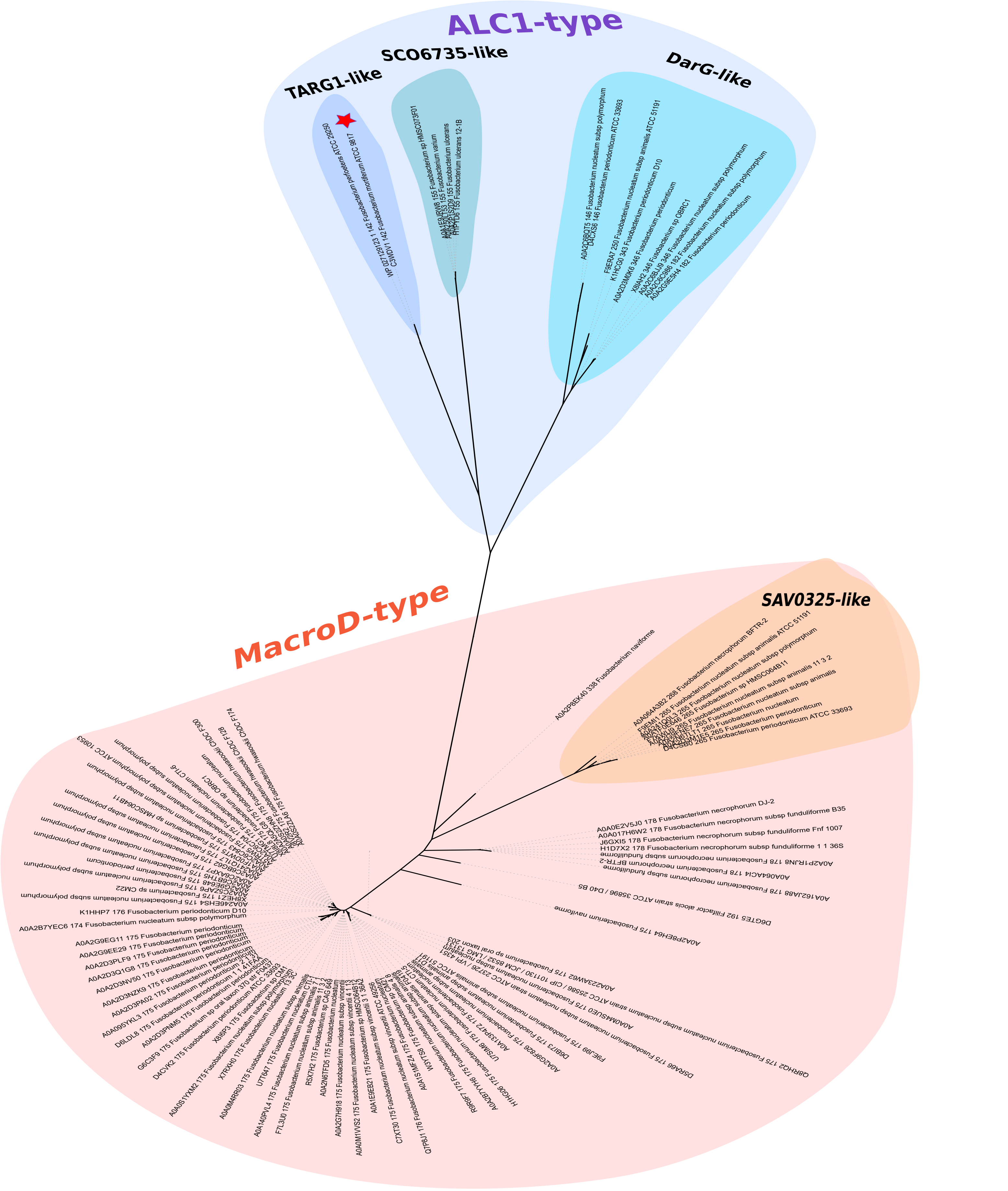

Supplementary Figure S2. Phylogenic tree of *Fusobacterium* macrodomains. The neihbour-joining tree with the 94 sequences found in UniProt and NCBI databases was constructed using 1000 replicates. MacroD-type macrodomains (red) are most abundant, although ALC1-type macrodomain (blue) sequences are also found, including TARG1-, SCO6735- and DarG-like macrodomains, respectively. The *F. mortiferum* ATCC 9817 TARG1 macrodomain is marked with a red star.

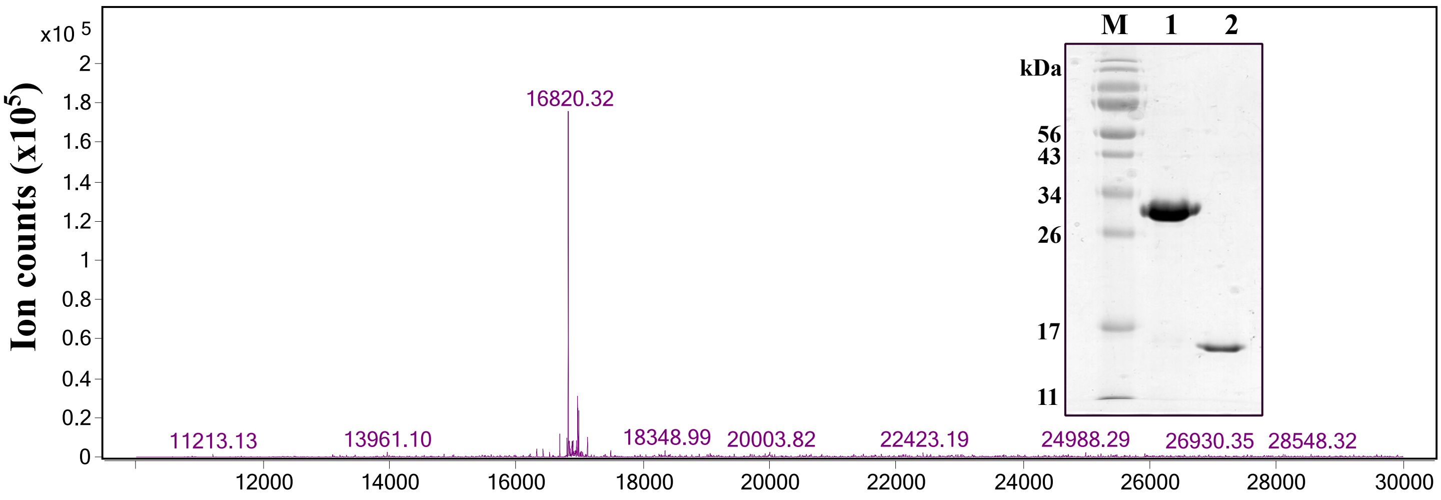

Supplementary Figure S3. Purification of FmTARG1. Mass spectra of purified FmTARG1 showed a molecular mass of 16820.32 Da. Insert. SDS-PAGE (15 %) gel of FmTARG1 purification process. Molecular mass standards (M) (kDa) are indicated in the left margin (EZ-Run^{TM} Pre-Stained *Rec* Protein ladder, Fisher). AFV-FmTARG1 and FmTARG1 proteins are shown as a band of about 30 kDa (lane 1) and 16 kDa (lane 2), respectively.

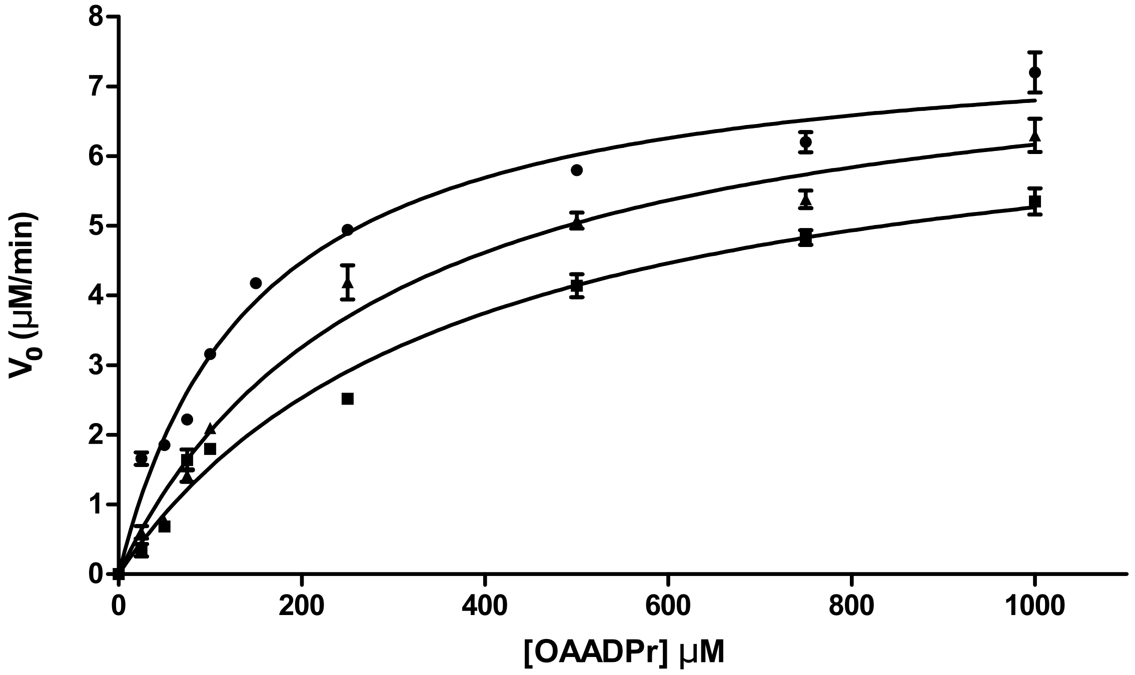

Supplementary Figure S4. FmTARG1 activity towards O-acetyl-ADP-ribose. The steady-state kinetic of FmTARG1 (●) was also compared with hTARG1 (■) at a fixed enzyme concentration (0.2 µM). The inhibitory effect by ADPr on the deacetylation reaction of OAADPr was studied at 200 µM (▲).

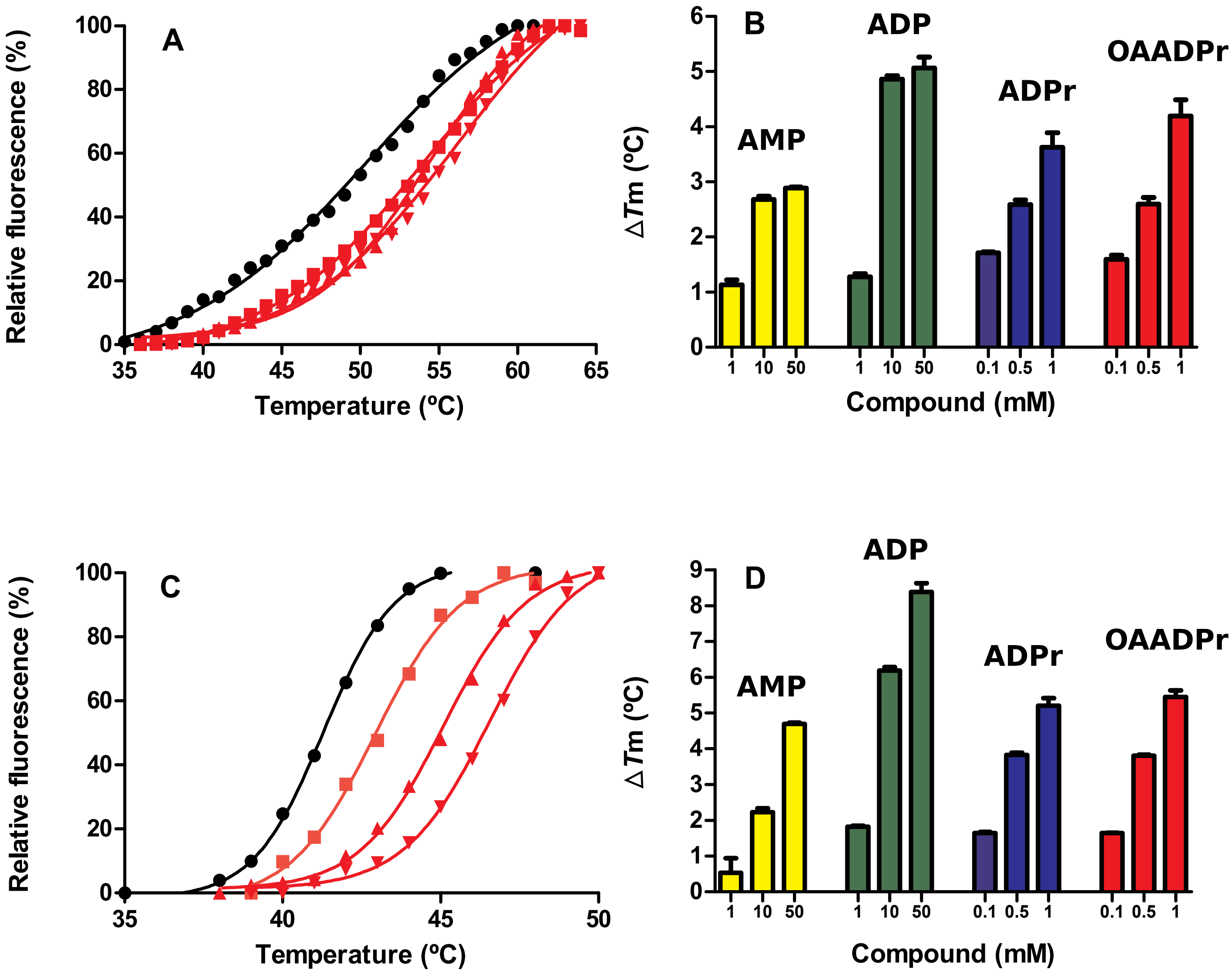

Supplementary Figure S5. Thermal shift assay of FmTARG1 and hTARG1. (**A**) Melting temperature curves of FmTARG1 in 50 mM sodium phosphate buffer pH 7.0 (●) and in the presence of different OAADPr concentrations: 0.1 mM (■), 0.5 mM (▲) 1 mM (▼). (**B**) Increment in melting temperature (Δ *T*m) of FmTARG1 in the presence of different ADPr analogues. The compound used were AMP and ADP at 1-50 mM, and ADPr and OAADPr at 0.1-1 mM. (**C**) Melting temperature curves of hTARG1. (**D**) Increment in melting temperature (Δ *T*m) of hTARG1 in the presence of different ADPr analogues. The conditions and concentrations used for hTARG1 were the same as for FmTARG1.

A

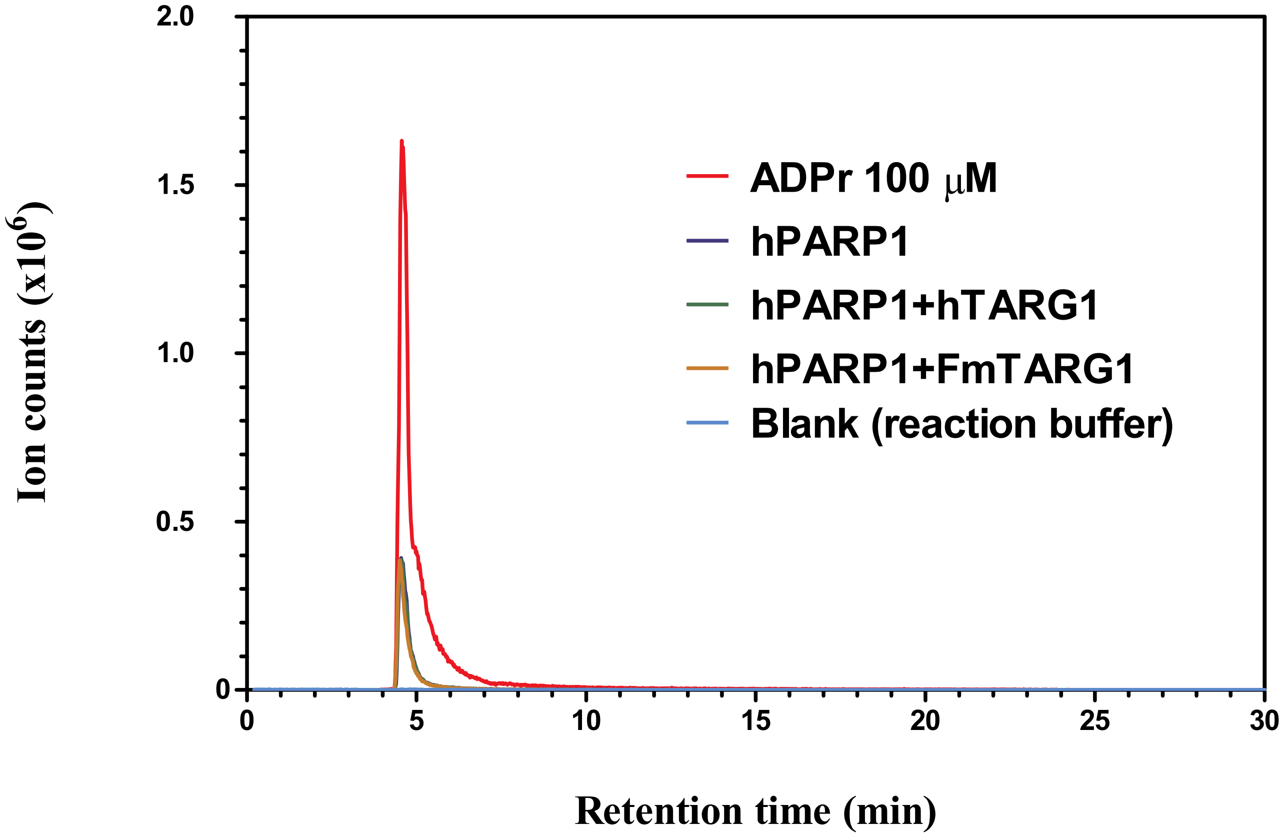

B

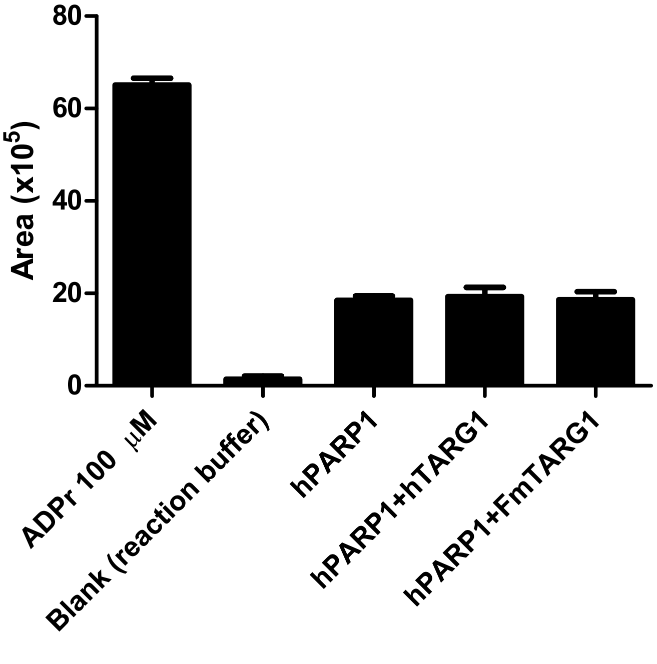

Supplementary Figure S6. Analysis of the reaction products of hTARG1 and FmTARG1 on poly(ADP-ribosyl)ated PARP1 by mass spectrometry. (**A**) Ion chromatograms of the monoisotopic mass of the ADP-ribose [M + H]+ adduct (560 m/z) obtained after the de-PARylation reactions catalyzed by FmTARG1 and hTARG1. (**B**) Area of mass 560.0 found in the different de-PARylation reactions. Error bars represent the standard deviation of three replicates. hPARP1 represents auto-PARylated hPARP1, whereas hPARP1+hTARG1 and hPARP1+FmTARG1 represent the de-PARylation reactions.
